# Experimental Evaluation and Machine Learning-Based Prediction of Laser Cutting Quality in FFF-Printed ABS Thermoplastics

**DOI:** 10.3390/polym17131728

**Published:** 2025-06-20

**Authors:** Gokhan Basar

**Affiliations:** Department of Industrial Engineering, Faculty of Engineering and Natural Sciences, Osmaniye Korkut Ata University, 80010 Osmaniye, Türkiye; gokhanbasar@osmaniye.edu.tr

**Keywords:** CO_2_ laser cutting, fused filament fabrication, ABS thermoplastics, surface roughness, kerf geometry, heat-affected zone, machine learning

## Abstract

Additive manufacturing, particularly Fused Filament Fabrication (FFF), provides notable advantages such as design flexibility and efficient material usage. However, components produced via FFF often exhibit suboptimal surface quality and dimensional inaccuracies. Acrylonitrile Butadiene Styrene (ABS), a widely used thermoplastic in FFF applications, commonly necessitates post-processing to enhance its surface finish and dimensional precision. This study investigates the effects of CO_2_ laser cutting on FFF-printed ABS plates, focusing on surface roughness, top and bottom kerf width, and bottom heat-affected zone. Forty-five experimental trials were conducted using different combinations of plate thickness, cutting speed, and laser power. Measurements were analysed statistically, and analysis of variance was applied to determine the significance of each parameter. To enhance prediction capabilities, seven machine learning models—comprising traditional (Linear Regression and Support Vector Regression), ensemble (Extreme Gradient Boosting and Random Forest), and deep learning algorithms (Long Short-Term Memory (LSTM), LSTM-Gated Recurrent Unit (LSTM-GRU), LSTM-Extreme Gradient Boosting (LSTM-XGBoost))—were developed and compared. Among these, the LSTM-GRU model achieved the highest predictive performance across all output metrics. Results show that cutting speed is the dominant factor affecting cutting quality, followed by laser power and thickness. The proposed experimental-computational approach enables accurate prediction of laser cutting outcomes, facilitating optimisation of post-processing strategies for 3D-printed ABS parts and contributing to improved precision and efficiency in polymer-based additive manufacturing.

## 1. Introduction

Additive manufacturing (AM), being another name for three-dimensional (3D) printing, has brought a new dawn in manufacturing with such options as design flexibility, material optimisation, and rapid prototyping [1]. This technique of manufacturing facilitates the deposition of material in layers, thus lessening material waste and allowing greater complexity in geometry, contrary to normal subtractive manufacturing techniques [2]. Not only does this manufacturing technique provide for higher material efficiency but it also facilitates design freedom, lightweight structures, and quick prototyping—the latter being extremely critical in aerospace, where weight reduction is crucial [3]; biomedical engineering, where patient-specific implants and tissue scaffolds are required [4]; automotive manufacturing, where customisation and iterative testing to speed development are required [5]; consumer electronics, which demands compact, intricate geometries [6]; and education, where the cheap costing of prototypes aids in practical learning [7]. Among the AM technologies, Fused Filament Fabrication (FFF) remains popular because of the affordability of the systems, easy operations, and working with a large variety of thermoplastic materials [8]. The working principle of FFF involves extruding thermoplastic filaments from a heated nozzle with successive layer deposits of the molten filament until completion of the final part. Over the last 15 years, an increasing number of engineering-grade thermoplastics have been employed in FFF to make truly functional parts that can endure both mechanical and thermal loadings in realistic applications [9]. Material-specific properties such as melt flow index, mechanical strength, and thermal behaviour consistently impose constraints, frequently hindering filament formulation and extrusion in FFF processes [10,11,12].

Acrylonitrile Butadiene Styrene (ABS) thermoplastics are engineering thermoplastics most widely used in FFF due to their favourable properties, such as high impact resistance, thermal stability, and excellent machinability; hence, they find application in enclosures, gears, automotive parts, and structural components [13]. However, FFF-printed parts from ABS suffer from dimensional inaccuracies and surface finish limitations owing to layer-by-layer deposition, warping, and nozzle resolutions [14]. Hence, post-processing techniques are used to further enhance the geometric fidelity and functional performance of printed parts; laser cutting is one such post-processing method that has gained much prominence over time [15]. Laser cutting offers a more precise and contactless method of trimming, shaping, and surface finishing [16].

From laser cutting, mainly CO_2_ laser cutting, a good finish to acrylic sheets is attained along with other non-metallic materials. Laser cutting uses much more precision than any other method, is less intensive on the mechanical stresses of materials, and produces perfectly clean-cut edges [17]. Laser cutting involves directing a high-power laser beam to melt, burn, or vaporise materials along a defined path [2]. In the case of 3D-printed polymers, it is often employed as a secondary finishing or customisation step after the initial fabrication of geometries. In particular, CO_2_ laser cutting is best suited for thermoplastics such as ABS because the substances absorb laser energy at that laser wavelength (10.6 μm), which leads to efficient energy transfer and smooth kerf formation [18]. This laser cutting technique can be used for everything from prototyping, packaging, to microfabrication, and decorative finishing applications in both industrial and research contexts [19].

Several authors have studied CO_2_ laser cutting of polymeric 3D-printed parts with the goal of improving the surface quality, kerf geometry, and dimensional accuracy. Kechagias et al. [20] conducted an extensive experimental study on CO_2_ laser cutting of FFF-printed ABS sheets, assessing four process parameters, namely stand-off distance, raster deposition angle, laser speed, and laser power, and their effects on kerf geometry and surface roughness. Cutting conditions were fully factorially designed, and regression analyses were performed to select the best cutting settings, demonstrating that dimensional accuracy and surface finish can be greatly improved by optimising the parameters. Similarly, Sabri et al. [21] scrutinised the CO_2_ laser cutting of PETG FFF plates using response surface methodology (RSM). They found that kerf widths on both upper and lower sides increased with the increase of laser power and the decrease of cutting speed, while the heat affected zone (HAZ) minimised when power was lower and speed was higher. Another relevant contribution by Moradi et al. [22] was the post-treatment of PLA parts by means of low-power CO_2_ lasers. Using central composite design, they studied the effect of focal plane position, power, and speed on kerf quality to optimise it to a state of minimal tapering and dimensional deviations. In a related study, Kechagias et al. [23] assessed the surface characteristics of 3D-printed PETG plates, employing factorial design and neural networks to model the relationship between laser cutting parameters and quality outputs. Their findings showed significantly improved surface roughness values compared to untreated FFF surfaces. Furthermore, Kechagias et al. [24] also studied surface quality in CO_2_ laser cutting of PLA parts and reported that cutting speed is a dominating factor in kerf angle formation. Meanwhile, both speed and power govern surface roughness. Their statistical treatments offered insightful leads for the parameter optimisation of laser cutting of biodegradable polymers. Whereas these findings were important milestones, the current literature remains insufficient for certain aspects, such as the CO_2_ laser cutting of ABS FFF-printed parts. Most studies focused on single materials (e.g., PLA or PETG) or examined limited parameters without evaluating multiple performance indicators simultaneously—such as surface roughness, top/bottom kerf width, and heat-affected zone (HAZ). Additionally, few studies explore machine learning for predictive modelling. To the author’s knowledge, only Kechagias et al. [20] provide a significant study on ABS, but their work has narrow parameter ranges and lacks predictive analytics. Consequently, extensive studies including various levels of thickness, laser powers, and speeds, along with employing machine learning for prediction and optimisation, are yet missing from the literature.

In this experimental setup, 3D-printed ABS plates ranging in thickness were laser cut using a CO_2_ laser for the three powers and the three speeds, thereby yielding 45 experimental settings. Each cut’s quality was evaluated based on surface roughness (Ra), kerf width at the top and bottom (Top KW, Bottom KW), and extent of heat-affected zone on the bottom (Bottom HAZ). The novelty of the study lies in the integration of a comprehensive experimental design with machine learning modelling. Different ML algorithms were used to predict performance indices from input parameters, and the prediction potential of each was rigorously evaluated. This mixed experimental–computational methodology thus gives insight from physical experimentation while deriving a powerful predictive capability from data, addressing a large knowledge gap. This study aims to optimise and predict how well CO_2_ laser cutting can be applied to FFF ABS parts. The research supports the development of enhanced post-processing techniques for 3D-printed polymers that can make these materials better suited for engineering uses by efficiently evaluating key process parameter influences and using machine learning models.

## 2. Materials and Methods

### 2.1. ABS Parts Manufacturing with Fused Filament Fabrication (FFF)

The 3D-printed samples in this study were made using ABS filament of 1.75 mm diameter supplied by the Filameon Co. (Kayseri, Türkiye). The material has good heat resistance, impact strength, and wear resistance, and thus may be used for functional purposes. It also has a high melt flow index, aiding the interlayer adhesion and nozzle life during printing. The technical data for ABS filament are given in Table 1.

**Figure 1:**
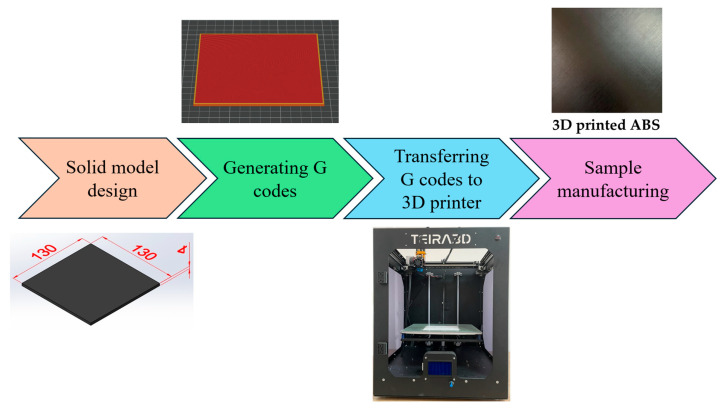
Manufacturing stages of ABS parts with a 3D printer.

Laser cutting samples, each having a surface area of 130 × 130 mm^2^ and thicknesses of 2, 2.5, 3, 3.5, and 4 mm, were designed using SolidWorks 2020 CAD software. G-codes were generated using PrusaSlicer 2.6.1, and samples were realised on an FFF-based TEIRA3D printer. The manufacturing process is shown schematically in Figure 1.

A preliminary experimental study was conducted to evaluate the 3D printing conditions needed to successfully fabricate ABS parts. The fixed parameters for the application were entered according to Table 2 into PrusaSlicer 2.6.1 slicing software.

### 2.2. CO_2_ Laser Cutting Process

Laser cutting experiments on 3D-printed ABS samples were conducted by means of a continuous-wave CO_2_ laser (LazerFix LF7010 Laser Cutting Machine; Konya, Türkiye) with a nominal power rating of 100 W, integrated with a three-axis CNC-controlled table. The nozzle-to-workpiece distance was maintained at 7 mm for all trials, which ensured good repeatability and precision. This distance was found to be optimal for keeping the laser beam’s focus sufficiently close to the molten material for convenient removal from the cutting zone. The compressed air was funnelled into the cutting zone to blow away the molten ABS from the cutting region and to keep the laser focusing optics free of dust and dirt that may be generated during the laser cutting process. Three sets of laser cutting parameters, representing the plate thickness, the cutting speed, and the laser power, were chosen for this study. These three parameters represented the main factors considered in this study, and each factor was evaluated at three different levels. The parameter ranges were chosen based on preliminary trials and are typical settings for laser cutting of thermoplastic materials such as ABS. Selected factors and their levels are summarised in Table 3.

**Figure 2:**
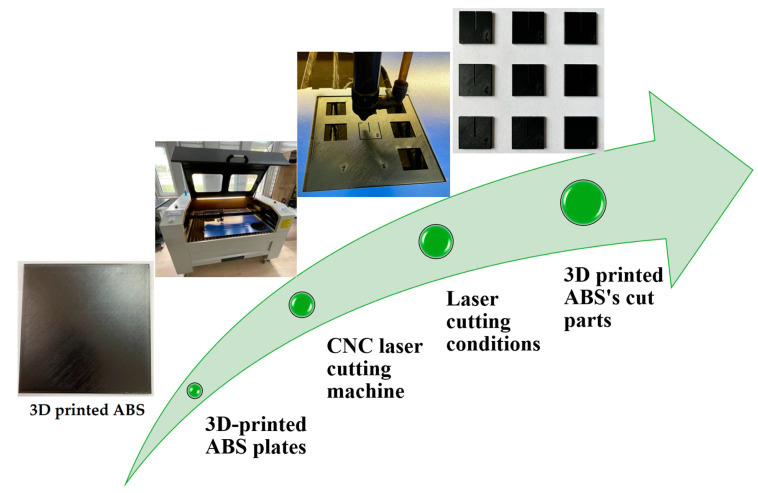
Sequencing CO_2_ laser cutting processes of 3D-printed ABS samples.

The 3D-printed ABS plates were secured to the cutting table with blue polypropylene material to prevent any movement during the laser cutting process. The geometric pattern consisted of nine square specimens measuring 25 mm × 25 mm each, cut from an initial square plate of 130 mm × 130 mm (Figure 2). To facilitate correct and consistent positioning, a straight guideline was drawn over 15 mm on the bigger plate, marking the squares to be cut. After the cut, each sample was subject to Ra measurement and KW measurement to find the effect of the cutting parameters on quality. These measurements were later used to make an inference—determining the best cutting parameters that would result in the smoothest surface finish with increased precision.

### 2.3. Measurement of Performance Indicators

**Figure 3:**
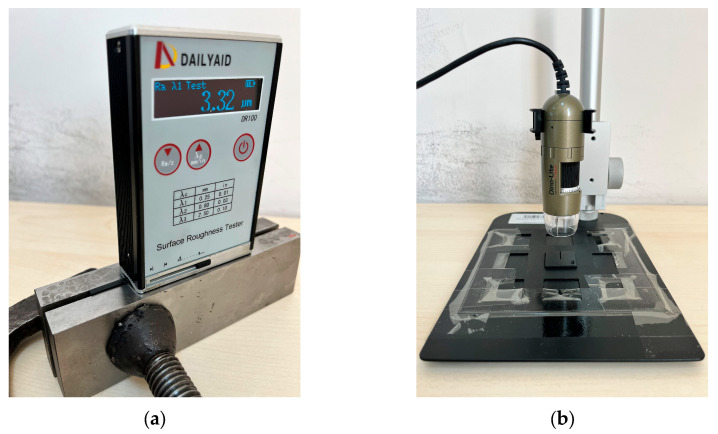
Measurement devices; (**a**) surface roughness device, (**b**) digital microscope.

After CO_2_ laser cutting applications on 3D-printed ABS plates, the considered laser parameters were cutting speed and pulse frequency. Ra, Top KW, Bottom KW, and Bottom HAZ were selected as the process outputs in laser cutting. The measurements were performed to observe the effect of cutting parameters on output performance criteria. During laser cutting, samples were cut in the form of squares with lengths of 25 mm. For Ra measurements, these samples were clamped in between two metal blocks. Parallel alignment was maintained by keeping the surface of the cut sample on the same axis as the metal blocks to allow the surface roughness tester to be placed properly. The purpose here is to make parallel contact between the surface roughness measurement probe and the cut sample surface. Thus, more reliable and accurate measurements were obtained [17]. Ra was measured on three edges of each cut sample; on each edge, three measurements were taken, and the results averaged over a total of nine measurements. The Ra values were obtained using a DAILYAID DR100 surface roughness tester (Beijing Dailyaid Measuring & Control Ltd., Beijing, China). To measure the Top KW, Bottom KW, and Bottom HAZ on the cut sample, a slit was created on the cut sample. This slit was imaged using a Dino-Lite AM4113T digital optical microscope (AnMo Electronics Corporation, New Taipei City, Taiwan), and precise measurements were performed on these images by using Dino Capture 2.0 software. Four measurements were performed for the Top KW, and six measurements were taken for both Bottom KW and Bottom HAZ; average values were calculated. The devices used in the measurements are shown in Figure 3.

## 3. Machine Learning Models

**Figure 4:**
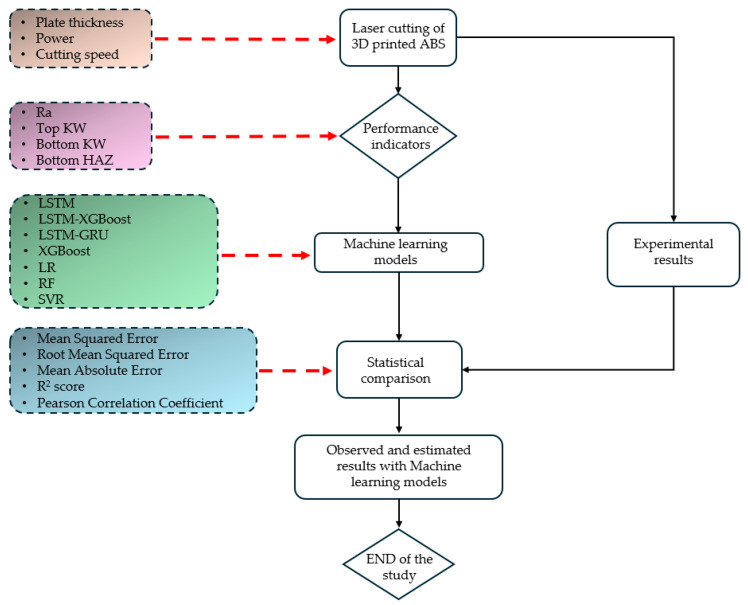
The flow chart of this study.

In this study, a comprehensive ML framework was established to predict laser cutting quality metrics. These metrics include Ra, Top KW, Bottom KW, and Bottom HAZ, which were estimated based on three key input parameters: plate thickness, laser power, and cutting speed. Initially, from the experimental dataset, the input and output variables were extricated, and then each output variable was modelled separately. The partitioning of the dataset into a training and testing set was accomplished with an 80-20 split ratio. Both features and target variables were standardised with StandardScaler for numerical stability in model training [25]. Seven models were developed and analysed to assess the model performance. These models fell into three categories: (1) deep learning: LSTM, LSTM-GRU, and LSTM-XGBoost; (2) ensemble learning: XGBoost and Random Forest; and (3) traditional regression: Linear Regression (LR) and Support Vector Regression (SVR). Deep learning models were constructed using TensorFlow’s Keras API whereby the LSTM and GRU layers were either stacked or combined. These would then be trained with the Adam optimiser at a learning rate of 0.001. The traditional ML models, including XGBoost, SVR, RF, and LR, were executed using scikit-learn and XGBoost implementations along with relevant hyperparameter tuning [25]. Here, each model was fitted on the standardised training dataset and scored on unscaled test targets for different performance metrics: MSE, RMSE, MAE, R-squared, and Pearson’s correlation. To further assist interpretations and comparisons, scatter plots of predicted versus observed values for each target variable were also prepared with uniform plot aesthetics such as marker size, colour, and font size. The entire computation pipeline and visualisations were carried out in Python using a single software stack comprising pandas, NumPy, scikit-learn, TensorFlow, XGBoost, and Matplotlib. The entire computation pipeline and visualisations were conducted in Python (v3.11.13) using a unified software stack comprising Pandas (v2.2.2), NumPy (v2.0.2), Scikit-learn (v1.6.1), TensorFlow (v2.18.0), XGBoost (v2.1.4), and Matplotlib (v3.10.0). The flowchart showing the machine learning model development and evaluation process followed in this study is presented in Figure 4.

### 3.1. Long Short-Term Memory (LSTM)

The LSTM model is a type of recurrent neural network designed to capture temporal dependencies using memory cells and gating mechanisms [26]. In this study, a single-layer LSTM network was employed to directly predict the target value from three-dimensional input data. Equation (1) produces the hidden state $h_{t}$ from input $X_{t}$, and Equation (2) maps $h_{t}$ to the output $\hat{y}$.

(1) $$h_{t}=LSTM\left(X_{t},h_{t-1},c_{t-1}\right)$$

(2) $$\hat{y}=W\cdot h_{t}+b$$

where $X_{t}$ is the input vector at time *t*. $h_{t}$ is the hidden state at time *t*. $c_{t}$ is the cell state at time *t*. W is the weight matrix of the output layer. b is the bias term.

### 3.2. LSTM-Gated Recurrent Unit (LSTM-GRU)

This hybrid model first employs a LSTM layer to learn temporal features, followed by a GRU layer that compresses sequential information for the final prediction [27]. The cascade architecture enhances temporal feature extraction by combining both memory mechanisms. Equation (3) captures temporal features as $h_{t}^{\left(LSTM\right)}$, while Equation (4) condenses them into $h_{t}^{\left(GRU\right)}$ via a GRU and then linearly projects this vector to the final prediction $\hat{y}$.

(3) $$h_{t}^{\left(LSTM\right)}=LSTM\left(X_{t}\right)$$

(4) $$h_{t}^{\left(GRU\right)}=GRU\left(h_{t}^{\left(LSTM\right)}\right)\Rightarrow \hat{y}=W\cdot h_{t}^{\left(GRU\right)}+b$$

$X_{t}$ is the input at time *t*. $h_{t}^{\left(LSTM\right)}$ is the output from the LSTM. $h_{t}^{\left(GRU\right)}$ is the output from the GRU. W and b are the output layer weights and bias.

### 3.3. LSTM-Extreme Gradient Boosting (LSTM-XGBoost)

In this architecture, the LSTM acts as a feature extractor, and the resulting temporal representations are passed to an XGBoost model for final regression [28]. This hybrid framework combines deep sequential modelling with tree-based gradient boosting. Equation (5) extracts temporal features as $h_{t}$ via a LSTM, and Equation (6) feeds $h_{t}$ into XGBoost to yield the final prediction $\hat{y}$.

(5) $$h_{t}=LSTM\left(X_{t}\right)$$

(6) $$\hat{y}=XGBoost\left(h_{t}\right)$$

where $X_{t}$ is the input sequence. $h_{t}$ is the LSTM feature output. $\hat{y}$ is the predicted output.

### 3.4. Extreme Gradient Boosting (XGBoost)

XGBoost is an ensemble learning algorithm based on additive tree models that minimise a loss function using gradient descent. It builds a series of weak learners (decision trees) sequentially to optimise predictive performance. Equation (7) states that XGBoost predicts ${\hat{y}}_{i}$ by summing the outputs of K decision-tree functions ${\;f}_{k}$, i.e., an additive ensemble over the input $x_{i}$ [29].

(7) $${\hat{y}}_{i}=\sum_{k=1}^{K}f_{k}\left(x_{i}\right),\;{\;f}_{k}\epsilon F$$

where $x_{i}$ is the feature vector for observation *i*. ${\hat{y}}_{i}$ is the predicted output. $f_{k}$ is the k-th weak learner (tree). K is the number of trees. F is a space of regression trees.

### 3.5. Linear Regression (LR)

LR models the relationship between independent variables and a continuous dependent variable using a linear function. The coefficients are estimated using the least squares method to minimise prediction error [30]. Equation (8) predicts $\hat{y}$ as the intercept $\beta_{0}$ plus the weighted sum of inputs $x_{j}$ with coefficients $\beta_{j}$.

(8) $$\hat{y}=\beta_{0}+\sum_{j=1}^{n}\beta_{j}x_{j}$$

where $x_{j}$ is the j-th input variable. $\beta_{j}$ is the regression coefficient. $\beta_{0}$ is the intercept. $\hat{y}$ is the predicted output.

### 3.6. Random Forest (RF)

RF is an ensemble method that aggregates the predictions of multiple decision trees trained on different subsets of the data. The final output is determined by averaging the predictions of all individual trees [31]. Equation (9) computes the RF prediction $\hat{y}$ as the means of the T decision-tree outputs $f_{t}\left(x\right)$.

(9) $$\hat{y}=\frac{1}{T}\sum_{t=1}^{T}f_{t}\left(x\right)$$

where x is the input vector. $f_{t}$ is the prediction of the *t*-th decision tree. T is the total number of trees. $\hat{y}$ is the predicted value.

### 3.7. Support Vector Regression (SVR)

SVR performs regression by mapping input data into a high-dimensional space using kernel functions and fitting a linear function within an ε-insensitive tube. It aims to find a function that deviates from the actual targets by at most ε [32]. Equation (10) predicts $\hat{y}\left(x\right)$ by adding the bias b to the kernel-weighted sum of support vector contributions $\left(\alpha_{i}-\alpha_{i}^{*}\right)K\left(x_{i},x\right)$.

(10) $$\hat{y}\left(x\right)=\sum_{i=1}^{n}\left(\alpha_{i}-\alpha_{i}^{*}\right)K\left(x_{i},x\right)+b$$

where $x_{i}$ is the support vector. $\alpha_{i},\alpha_{i}^{*}$ are the Lagrange multipliers. $K\left(x_{i},x\right)$ is the kernel function (e.g., RBF). b is the bias term. $\hat{y}$ is the predicted output.

### 3.8. Evaluation Metrics

To evaluate the model’s performance, both error-based and statistical metrics were used. The Mean Squared Error (MSE) represents the average of the squared differences between the actual and predicted values [33]. The MSE is sensitive to the magnitude of the error and is calculated using Equation (11).

(11) $$MSE=\frac{1}{n}\sum_{i=1}^{n}{\left(y_{i}-{\hat{y}}_{i}\right)}^{2}$$

The Root Mean Squared Error (RMSE) is calculated by taking the square root of the MSE; the error unit is the same as that of the predicted variable. The RMSE is computed using Equation (12).

(12) $$RMSE=\sqrt{\frac{1}{n}\sum_{i=1}^{n}{\left(y_{i}-{\hat{y}}_{i}\right)}^{2}}$$

The Mean Absolute Error (MAE) is the average of the absolute differences between actual and predicted values; it is easy to interpret and more robust to outliers [34]. The characteristic equation of the MAE is given by Equation (13).

(13) $$MAE=\frac{1}{n}\sum_{i=1}^{n}\left|y_{i}-{\hat{y}}_{i}\right|$$

The determination coefficient (R^2^) indicates the explanatory power of the model over the dependent variable. Values close to 1 represent a better fit. R^2^ is calculated using Equation (14).

(14) $$R^{2}=1-\frac{\sum_{i=1}^{n}{\left(y_{i}-{\hat{y}}_{i}\right)}^{2}}{\sum_{i=1}^{n}{\left(y_{i}-\bar{y}\right)}^{2}}$$

The Pearson Correlation Coefficient (Pearson), given in Equation (15), measures the degree of linear relationship between the actual and predicted values [35]. Its value ranges from [−1, +1], where values close to +1 indicate a strong positive correlation.

(15) $$Pearson=\frac{\sum_{i=1}^{n}\left(y_{i}-\bar{y}\right)\left({\hat{y}}_{i}-\bar{\hat{y}}\right)}{\sqrt{\sum_{i=1}^{n}{\left(y_{i}-\bar{y}\right)}^{2}}\sqrt{\sum_{i=1}^{n}{\left({\hat{y}}_{i}-\bar{\hat{y}}\right)}^{2}}}$$

Here, $y_{i}$ represents the actual value of the ith sample; ${\hat{y}}_{i}\;$ denotes the predicted value of the ith sample by the model; $\bar{y}$ is the meaning of the actual values; $\bar{\hat{y}}$ is the mean of the predicted values; and n denotes the total number of samples.

## 4. Results and Discussion

### 4.1. Assessment of Laser Cutting Performance Indicators

This subsection deals with interpreting the laser-cutting performance index values obtained from experiments carried out with the CO_2_ laser to evaluate the cutting performance of 3D-printed ABS materials. In other words, detailed analyses are presented for the following parameters: (1) surface roughness, Ra (µm); (2) top kerf width (Top KW, mm); (3) bottom kerf width (Bottom KW, mm); and (4) bottom heat-affected zone (Bottom HAZ, mm).

#### 4.1.1. Surface Roughness

**Figure 5:**
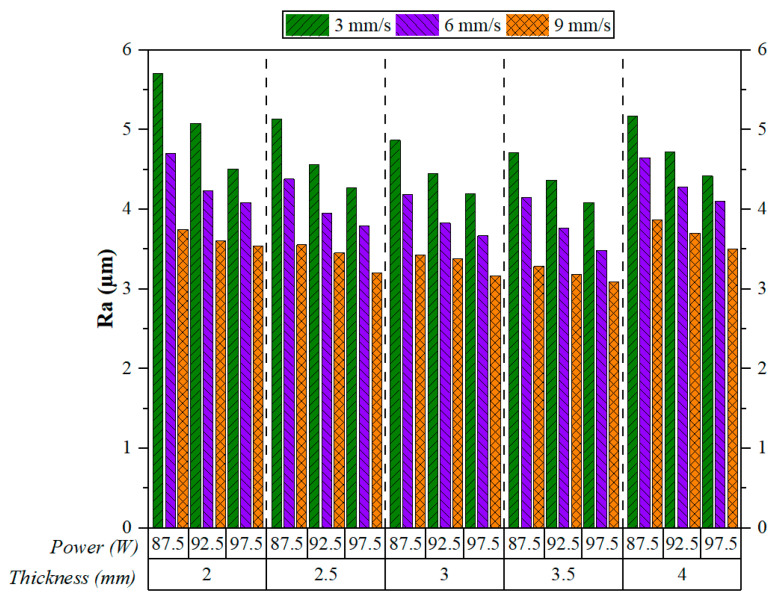
Effect of cutting speeds and cutting parameters on Ra.

The Ra values, according to Figure 5, are those attended to in CO_2_ laser cutting experiments on 3D-printed ABS plates of varying thicknesses under different laser power and speed values. On a general note, while the increasing cutting speed decreased Ra, this remained true irrespective of material thickness or laser power. Kechagias et al. found a similar result, which supported the observation that cutting speed very much tended to improve surface quality [36]. Most of the time, the Ra values would be the least at 9 mm/s, whereas at 3 mm/s, the Ra values would be the highest, pointing unequivocally in favour of higher cutting speeds for smoother surface finishes. The most pronounced Ra value was observed at 2 mm thickness, a laser power of 87.5 W, and a cutting speed of 3 mm/s.

Across all thickness regimes, the effect of cutting speed is much more dominant than that of laser power on the Ra. For instance, at a 2 mm thickness, the Ra drops from above 5 µm at 3 mm/s to below 4 µm at 9 mm/s, regardless of the changes in power. This observation is consistent for all thicknesses between 2 mm and 4 mm, hence solidifying that cutting speed plays a greater role in reducing surface irregularities. Laser power, on the other hand, does bear an influence over Ra, but to a lesser extent. However, for each speed, there is a slight decrease in Ra with an increase in power, although this is far from as important as what speed causes.

The combination of high speed (9 mm/s) and high power (97.5 W) produces, in general, the lowest Ra values for all thicknesses. In addition, plate thickness appears to moderately exert some effect on Ra. Thicker plates (3.5–4 mm) tend to have relatively stable Ra values, while thinner ones (2 mm) show slightly higher roughness, especially at lower speeds. This could be due to heat accumulation and the smaller mass of material available to dissipate thermal energy during cutting. Tamrin et al. acknowledged that heat accumulation should be controlled, and proper cutting parameters should be chosen to yield effective CO_2_ laser cutting of thermoplastics; when these are controlled, thermal damages are kept to a minimum and good quality is achieved [37]. As thickness increases, Ra decreases until reaching 3.5 mm; however, when the thickness becomes 4 mm, Ra sees a slight increase. In summary, the results indicate that higher cutting speeds significantly improve surface quality, with laser power and thickness having secondary but noticeable effects.

**Figure 6:**
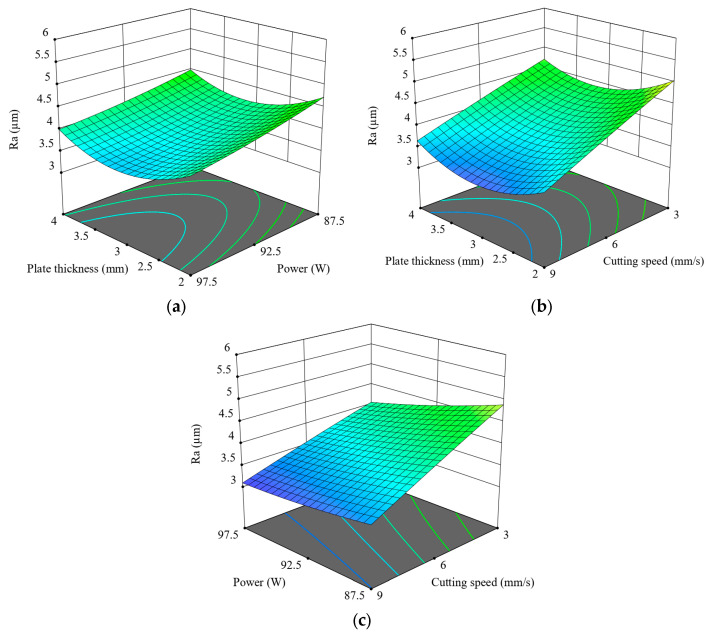
Three-dimensional surface plots for the effect of cutting parameters on Ra; (**a**) plate thickness–power, (**b**) plate thickness–cutting speed, (**c**) power–cutting speed.

Figure 6 consists of surface plots for the study of the combined effects of key process parameters on the Ra value during CO_2_ laser cutting of 3D-printed ABS parts. Figure 6a shows the effect of laser power and plate thickness on Ra value. It is evident that while an increase in laser power generally decreases Ra, especially at lower plate thicknesses, increasing surface quality with more energy input, this effect is somewhat diminished at greater thickness levels (3.5–4 mm), implying lesser penetration of energy or heat tending to dissipate. The effect of cutting speed and plate thickness on Ra values, shown in Figure 6b, indicates that increasing the cutting speed decreases Ra values at every thickness level, confirming that higher cutting speeds minimise thermal buildup and thus reduce the irregularity of the surface [38]. The minimum Ra values were obtained at the highest speed of 9 mm/s combined with a moderate thickness of 2.5–3 mm. Figure 6c shows that combined effects of laser power and cutting speed indicate the best Ra values with high power and high speed. In general, cutting speed is dominant, followed by laser power, while plate thickness is of moderate influence, the effect depending on the context. These findings align with general expectations and highlight the importance of parameter synergy in achieving optimal post-processing quality for FFF-printed thermoplastics.

**Figure 7:**
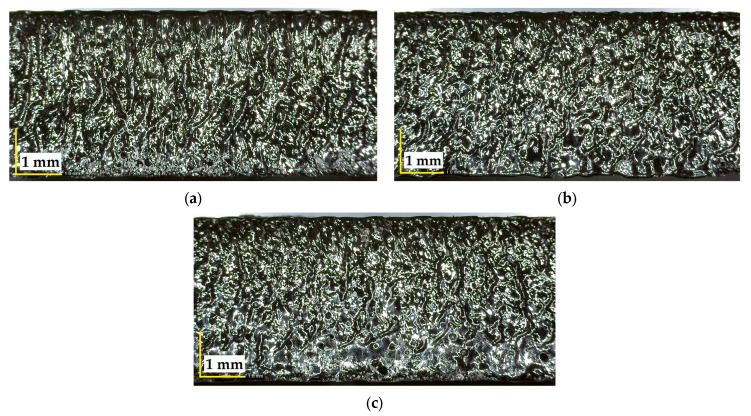
Surfaces cut at 87.5 W power, 3.5 mm plate thickness and different cutting speeds: (**a**) 3 mm/s, (**b**) 6 mm/s, (**c**) 9 mm/s.

Figure 7 illustrates that increasing the cutting speed from 3 mm/s to 9 mm/s at a constant power of 87.5 W and 3.5 mm plate thickness results in visibly smoother and more uniform surface textures, indicating improved surface quality at higher speeds.

#### 4.1.2. Top Kerf Width

**Figure 8:**
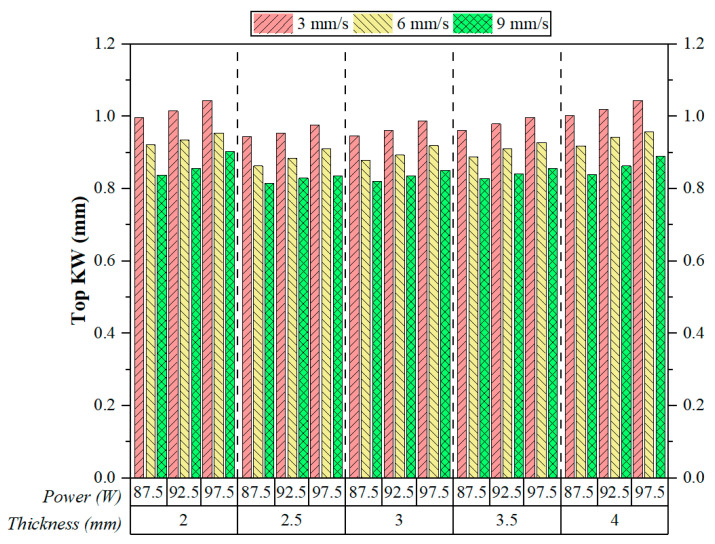
Effect of cutting speeds and cutting parameters on Top KW.

The experimental results of the KW determination with the CO_2_ laser are presented in Figure 8. The results show the effects of the factors, both individual and combined, namely, the cutting speed, laser power, and thickness of the material, on the kerf geometry at the upper edge of the cut. Through all experimental conditions, a very clear inverse relationship could be observed between cutting speed and Top KW. More specifically, Top KW values narrowed consistently with increased cutting speeds, thus indicating better dimensional accuracy. An analogous statement was presented by Kechagias et al., who stated that increasing the cutting speed leads to a reduction in the KW [36]. For instance, Top KW at 9 mm/s (green bars) were always smaller than at 6 mm/s (yellow bars) or 3 mm/s (red bars) for all thickness and power combinations. This can be explained by the reduced thermal diffusion time at higher cutting speeds, which limits the time available for material melting and lateral heat propagation, thereby preventing further kerf enlargement at the surface.

As expected, laser power had a somewhat direct effect on Top KW. At each cutting speed, the Top KW tends to increase a little as the power level goes up due to more energy being introduced into the system, which results in deeper penetration and wider molten zones. Sabri et al. reached a similar conclusion, where an increase in laser power leads to an increase in the Top KW [21]. However, at high cutting speeds, that effect was somewhat diminished, thus suggesting that cutting speed plays a more dominant role than laser power in affecting the thermal–material interaction generated during the cutting process. The effect of thickness was rather subtle in relation to Top KW. As thickness increased from 2 to 2.5 mm, Top KW somewhat decreased, whereas in the range from 2.5 to 4 mm, Top KW gradually increased. This could be accounted for by the fact that the thicker plates generally have increased thermal inertia that may result in heat being accumulated near the surface and thereby enhancing kerf expansion.

Moghadasi et al. [39] demonstrated that increased thermal inertia in thick thermoplastic plates during CO_2_ laser cutting leads to considerable surface heat accumulation. This accumulation significantly contributes to kerf widening and reduced cut quality, highlighting the need for optimised cutting parameters and effective cooling strategies. However, the variation was not significant, indicating a secondary effect of thickness in determining Top KW under the present experimental setup. One standout observation, regardless of material thickness, is that Top KW increases steadily with a low cutting speed (3 mm/s) and high laser power (97.5 W) level. On the other end of the spectrum, the most favourable kerf geometry—characterised by narrow and uniform cuts—was observed at a high cutting speed (9 mm/s) combined with moderate-to-high power settings, regardless of material thickness. The results hereby emphasise balancing the thermal energy input and interaction time to generate dimensionally accurate cuts with fine quality in the post-processing of FFF-manufactured ABS components.

**Figure 9:**
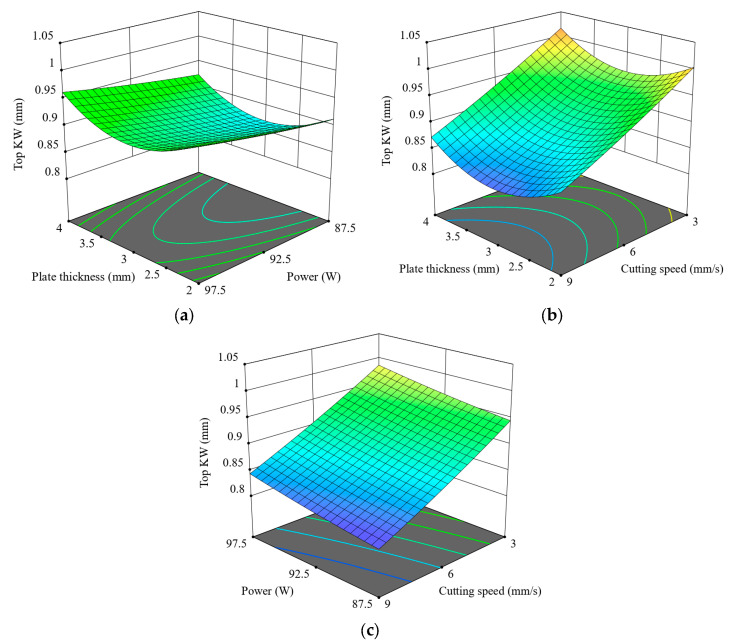
Three-dimensional surface plots for the effect of cutting parameters on Top KW; (**a**) plate thickness–power, (**b**) plate thickness–cutting speed, (**c**) power–cutting speed.

Figure 9 shows the combined effects of plate thickness, laser power, and cutting speed over Top KW during CO_2_ laser cutting of 3D-printed ABS. Observing Figure 9a, it can be seen that Top KW rises slightly with increasing laser power at lower thicknesses, but this effect tapers off with increasing thickness, possibly due to diminished energy penetration. Figure 9b portrays cutting speed and thickness exerting a much stronger influence: Top KW shrinks markedly with an increase in cutting speed for every thickness level, again marking the dominance of cutting speed in the minimisation of KW. The lowest Top KW values are obtained at high cutting speed (9 mm/s) combined with medium thickness (2.5–3 mm). Looking at Figure 9c, the interpretation is that a higher speed will yield a narrower KW for every level of power; in addition, lower powers may have a positive influence if combined with higher speeds for more controlled and precise kerf formation. Overall, cutting speed is the most powerful parameter in reducing Top KW, followed by laser power, and plate thickness comes somewhere in between, dependent on the situation. These 3D plots clearly illustrate the critical importance of multi-parameter optimisation for achieving dimensional accuracy in the laser post-processing of FFF-manufactured ABS parts.

**Figure 10:**
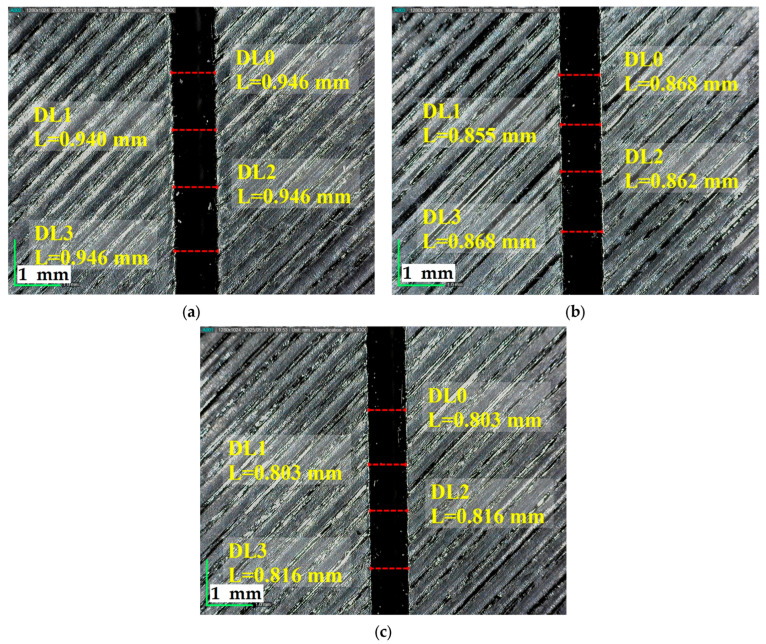
A total of 90 W power, 2.5 mm plate thickness, and Top KW at different cutting speeds; (**a**) 3 mm/s, (**b**) 6 mm/s, (**c**) 9 mm/s.

Figure 10 demonstrates that as the cutting speed increases from 3 mm/s to 9 mm/s at a constant power of 90 W and 2.5 mm plate thickness, Top KW consistently decreases, indicating enhanced cutting precision at higher speeds.

#### 4.1.3. Bottom Kerf Width

**Figure 11:**
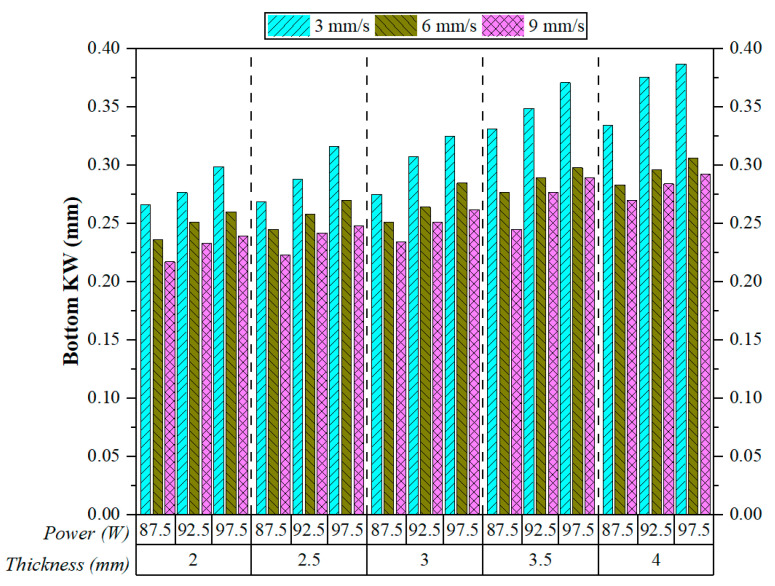
Effect of cutting speeds and cutting parameters on Bottom KW.

Figure 11 presents the measured Bottom KW values under various laser cutting parameters, including cutting speed, laser power, and material thickness. The figure clearly demonstrates a strong inverse relationship between cutting speed and Bottom KW. For all combinations of laser power and thickness, increasing the cutting speed from 3 mm/s to 9 mm/s consistently reduced the width of the kerf at the bottom edge of the cut. This trend was also observed in the study conducted by Sabri et al. [21]. This observation aligns with the physical understanding that higher cutting speeds reduce the thermal interaction time between the laser beam and the material, thereby limiting lateral heat propagation and the expansion of the melt pool at the bottom layer.

Among all experimental conditions, the largest Bottom KW values were recorded at the lowest speed setting (3 mm/s), particularly for thicker plates (3.5 mm and 4 mm) and higher laser power (97.5 W). For example, at 4 mm thickness and 97.5 W, Bottom KW reached nearly 0.38 mm at 3 mm/s, whereas it dropped below 0.30 mm at 9 mm/s. This reduction emphasises the critical role of cutting speed in mitigating thermal diffusion effects that would otherwise contribute to kerf widening at the bottom surface. The relatively poor performance at lower speeds can be attributed to prolonged energy exposure, which intensifies heat accumulation and causes excessive downward material melting. This behaviour was also reported in the previous works of Basar et al. [40,41]. Laser power also exhibits a direct influence on Bottom KW, though less prominent than the effect of cutting speed. At a constant speed, Bottom KW values gradually increase with rising power levels. This finding agrees with another study conducted by Kechagias et al. [42]. The explanation lies in the greater energy input at higher powers, which results in larger melting volumes and, consequently, wider KW. However, at higher cutting speeds, the widening effect of power becomes less significant, suggesting that increased speeds help mitigate the influence of power by limiting thermal penetration depth and associated material deformation.

Material thickness also contributes to Bottom KW variation, particularly under low-speed and high-power conditions. As plate thickness increases from 2 mm to 4 mm, Bottom KW values tend to rise. This behaviour can be attributed to the increased thermal inertia of thicker materials, which restricts heat dissipation and leads to greater heat accumulation near the bottom cutting edge [43]. As a result, the molten material cannot be efficiently expelled, leading to a broader kerf. In summary, cutting speed is the dominant parameter affecting Bottom KW, with higher speeds significantly enhancing cut precision at the bottom surface. Laser power has a secondary but noticeable effect, where higher power levels lead to wider kerfs. Material thickness plays a contextual role, particularly when cutting parameters are not optimally configured.

**Figure 12:**
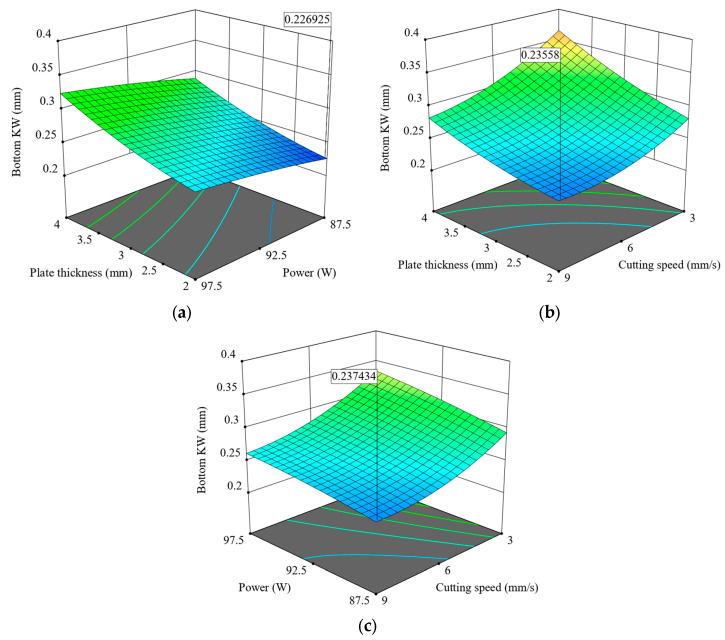
Three-dimensional surface plots for the effect of cutting parameters on Bottom KW; (**a**) plate thickness–power, (**b**) plate thickness–cutting speed, (**c**) power–cutting speed.

Figure 12 presents 3D surface diagrams showing combined effects of cutting variables on Bottom KW in CO_2_ laser cutting of 3D-printed ABS parts. In plot (Figure 12a), Bottom KW increases with increasing plate thickness or laser power; in other words, thicker plates and higher power promote greater kerf widening at the bottom surface. In plot (Figure 12b), Bottom KW decreases almost monotonically with cutting speed regardless of material thickness, clearly establishing the dominant role of speed in limiting heat diffusion and the spread of molten material. Thus, Bottom KW is minimum at high cutting speed and low to moderate thickness levels. It is also clear from Figure 12c that increasing cutting speed will reduce Bottom KW for various power levels, while power would slightly oppose this effect. Yet, the curvature of the surface is rather mild, indicating that cutting speed measures up as the more potent factor when compared to laser power in this scenario. Summarising, these plots show that minimising the Bottom KW calls for a compromise among the requisite variables, such as favouring largely the high cutting speed with moderate thickness and control over power.

**Figure 13:**
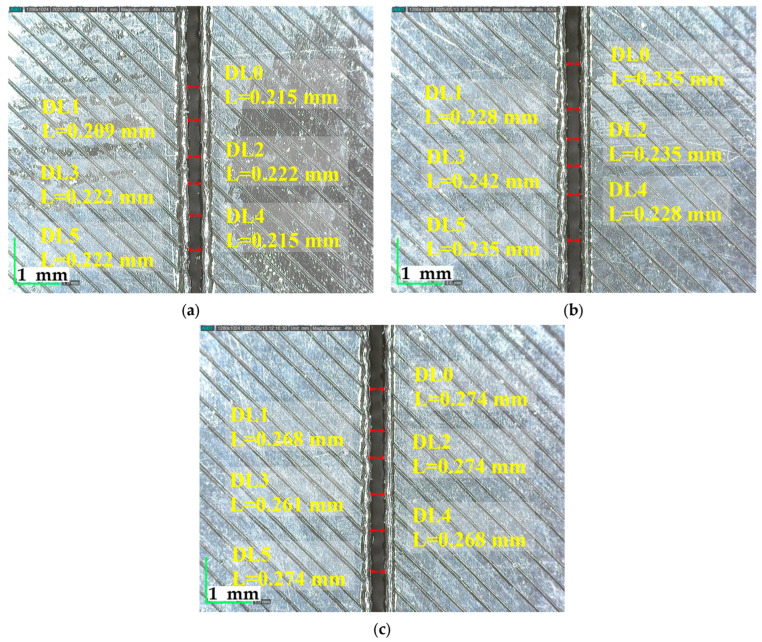
A total of 90 W power, 9 mm/s cutting speed, and Bottom KW at different cutting speeds; (**a**) 2 mm, (**b**) 3 mm, (**c**) 4 mm.

Figure 13 reveals that at a constant cutting speed of 9 mm/s and power of 90 W, Bottom KW increases with plate thickness, indicating a widening kerf profile as more thermal energy is absorbed by thicker materials.

#### 4.1.4. Bottom HAZ

**Figure 14:**
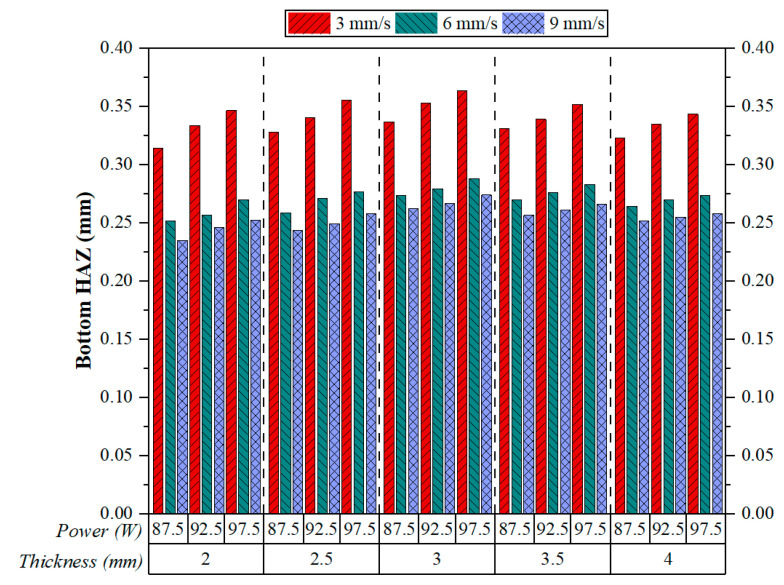
Effect of cutting speeds and cutting parameters on Bottom HAZ.

Figure 14 gives the measured Bottom HAZ values for 3D-printed ABS plates under different sets of cutting speed, laser power, and material thickness during CO_2_ laser cutting. A clearly defined and consistent trend emerges under all experimental conditions: the Bottom HAZ width increases drastically with the decrease in cutting speed, irrespective of the influence exerted by laser power or material thickness. This trend was also observed by Sabri et al. and lends strong credibility to the results [21]. At the lowest cutting speed, i.e., at 3 mm/s, the highest HAZ values were recorded. As for this, at the highest speed of 9 mm/s, the smallest values for the HAZs were recorded. Fundamentally, the inverse relation between cutting speed and Bottom HAZ can be explained by basic laser–material interaction concepts.

At low cutting speeds, the laser beam is permitted to remain longer on the surface of the material, leading to thermal penetration and heat accumulation. Kameyama et al. provided further elaboration on such phenomena that to practically suppress thermal effects during CO_2_ laser cutting of thermoplastics, it is important to use an advanced scheme, considering optimal process parameters, real-time monitoring, and material-dependent considerations [44]. If power and speed are optimally selected and combined with the use of assist gases, the amount of HAZ formation can be reduced, and the cutting can be qualified with lower thermal degradation. Longer exposure times at low speeds result in greater heat diffusion at the bottom surface. In contrast, high cutting speeds result in reduced interaction time and thus reduced thermal diffusion, corresponding to a tiny HAZ. Furthermore, laser power also has an effect, although less than speed. For any given thickness, when the laser power was increased from 87.5 to 97.5 W, a slight but easily noticeable increase in Bottom HAZ was found. This can, however, be attributed to an increase in energy being put into the process during operation at higher power, thus locally increasing the heat and causing it to further thermally conduct into the material [45]. Conversely, this effect will be largely reduced when high-speed cutting is again maintained, meaning that speed must be kept as the most important parameter for thermal control.

Thickness of the material also appeared to maintain a medium influence. For all speed and power combinations, an increment in thickness from 2 to 4 mm did not result in a meaningful change in HAZ size. This indicates that although thicker materials possess higher thermal inertia—and thus, perhaps, could have affected heat dissipation—the effect is secondary when it comes to the Bottom HAZ inside the cutting conditions employed by this study. Among all parameters set tested, the widest HAZ, around 0.36–0.37 mm, was found at 3 mm/s cutting, 97.5 W of power, and 3–3.5 mm thickness. However, the narrowed HAZ was always present at 9 mm/s, especially when the power level was moderate (92.5 W) and the material thickness thinner (2–2.5 mm). These results clearly highlight cutting speed as a major factor to be considered when minimising thermal damage during laser postprocessing of FFF-printed ABS components. It can thus be concluded that cutting speed dominates Bottom HAZ, laser power follows it as a secondary factor, and then material thickness assumes a limited role that depends on the context.

**Figure 15:**
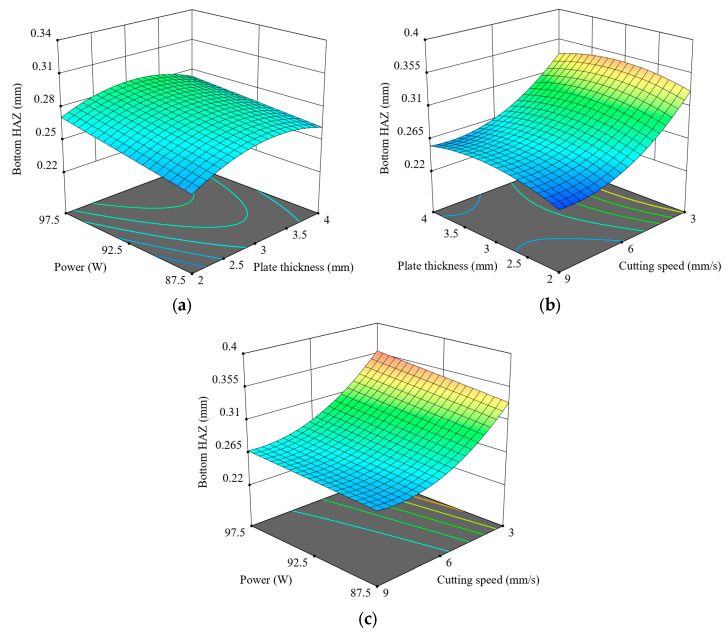
Three-dimensional surface plots for the effect of cutting parameters on Bottom HAZ; (**a**) plate thickness-power, (**b**) plate thickness-cutting speed, (**c**) power-cutting speed.

Figure 15 provides three-dimensional surface plots showing the effect of cutting parameters on Bottom HAZ. Plot (Figure 15a) suggests that Bottom HAZ tends to increase as one increases plate thickness and laser power, which is especially noticeable at high laser powers and for thick materials. Plot (Figure 15b) informs us that Bottom HAZ reduces almost linearly by increasing the cutting speed, no matter the thickness of the plate, which verifies the dominating role of speed in limiting thermal effects. The most severe reduction can be noted from 3 mm/s to 9 mm/s, primarily for thinner plates. Plot (Figure 15c) tells us that while power seems to have a slight increase effect on HAZ, the speed effect dominates entirely. High speed and low power are the conditions that minimise HAZ. In general, these surface plots convey that Bottom HAZ can be minimised by combining a high cutting speed with low to moderate power and thin to moderate thickness of the material. The results again stress that choosing parameters synergistically is paramount in controlling thermal effects under CO_2_ laser cutting of 3D-printed ABS.

**Figure 16:**
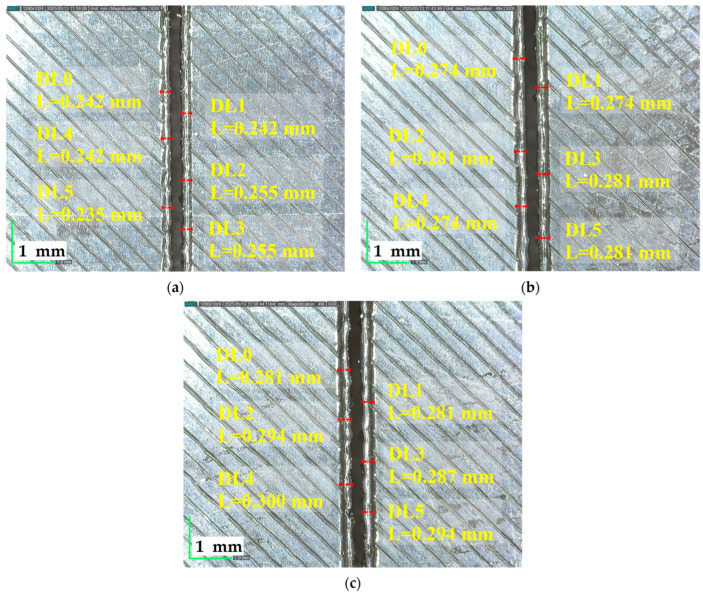
A total of 3.5 mm plate thickness, 9 mm/s cutting speed, Bottom HAZ at different powers; (**a**) 90 W, (**b**) 95 W, (**c**) 100 W.

Figure 16 shows that at a fixed cutting speed of 9 mm/s and 3.5 mm plate thickness, Bottom HAZ increases with laser power, indicating greater thermal penetration and energy input at higher power levels.

### 4.2. ANOVA Results

To statistically prove the influence of selected parameters on cutting quality, ANOVA was applied for each response variable: Ra, Top KW, Bottom KW, and Bottom HAZ. The ANOVA findings revealed the relative importance and contribution of plate thickness, laser power, and cutting speed in altering each quality metric in Table 4. All factors were statistically significant at the 95% confidence level (*p* < 0.001), ensuring the variations in cutting performance could be linked to the motion of the controlled process parameters. Among Ra factors, cutting speed was the most dominant parameter responsible for around 69.12% of the total variance, followed by laser power (14.45%) and plate thickness (12.25%). The residual error was quite low (4.18%), reinforcing the good adequacy of the model. The result fits well with the experimental observation of higher cutting speed always resulting in a lower value of Ra. The model features an R^2^ value of 95.82%, an adjusted R^2^ of 94.89%, and a predicted R^2^ of 93.47%, indicating the model has strong explanatory ability and predictive power. In the case of the Top KW parameter, cutting speed was likewise the most potent influence, explaining 81.52% of the observed variance. Laser power and plate thickness interceded with 6.43% and 10.99% of the variance, respectively. Coupled with this low error variance of only 1.05%, it can be assumed that the KW measured are both repeatable and accurate. An R^2^ of 98.95% was achieved by the statistical model for Top KW, with adjusted and predicted R^2^ of 98.71% and 98.35%, respectively, demonstrating its robustness in characterising the parameter effects on the kerf geometry at the top edge. For Bottom KW, the trend remained as before, with cutting speed exerting the maximum influence (47.29%), while plate thickness was next in line with 35.99% and then laser power at 11.56%. The error term had a moderate contribution (5.16%), which again favours the model’s sufficiency. It is interesting to observe how plate thickness exerts a considerable effect on Bottom KW relative to Top KW; this might be because of increased thermal inertia and the heat travelling downwards in thicker samples. By contrast, the model proved reliable with R^2^ being 94.84%, adjusted R^2^ of 93.70%, and predicted R^2^ of 91.94%. Again, for Bottom HAZ, cutting speed remained the overwhelmingly dominant factor, explaining 92.04% of the observed variance. Understandably, minor contributions were offered by laser power and plate thickness at 3.5% and 3.51%, respectively, while the error term remained negligible (0.95%). The enormous F-value of 1745.89 shows cutting speed’s critical role in reducing thermal damage. The model is supported by an R^2^ value of 99.05%, an adjusted R^2^ of 98.84%, and a predicted R^2^ of 95.52%, indicating exceptional predictive power with minimal risk of overfitting. In total, therefore, ANOVA strongly backs the experimental results and cites cutting speed as the most important process control on all performance indicators. Laser power and plate thickness certainly matter, but only secondarily and depending on circumstance.

### 4.3. Machine Learning Modelling Results

In this study, the effects of laser cutting parameters on cut quality were modelled using ML methods. Various hybrid and traditional algorithms were comparatively evaluated during the modelling process. The input parameters for the ML models were plate thickness, laser power, and cutting speed, while the output parameters were defined as Ra, Top KW, Bottom KW, and Bottom HAZ. The dataset was split into 80% for training and 20% for testing. Both input and output variables were standardised using the StandardScaler function. The LSTM model architecture employed a single-layer LSTM network, which, as shown in previous studies, offers a balance between simplicity and functionality, making it a practical choice for sequential data processing tasks despite sometimes lagging deeper architectures in predictive performance [46,47]. The ReLU activation function was used to introduce nonlinear, real-world experimental features into the model, as it is a widely adopted function that not only introduces nonlinearity but also helps mitigate the vanishing gradient problem, thereby facilitating the learning of complex patterns in deep learning architectures [48,49]. The model was optimised using the Adam optimiser (learning rate = 0.001), and the MSE was chosen as the loss function. The LSTM–XGBoost hybrid model consists of an LSTM layer with 32 neurons connected to an XGBoost layer with 16 neurons, also activated by ReLU. This hybrid was trained using the Adam optimiser (learning rate = 0.001). In the LSTM-GRU hybrid model, the sequence outputs of the 32-neuron LSTM layer were passed into a GRU layer with 32 neurons. The XGBoost algorithm based on gradient boosting was configured with 100 decision trees and a learning rate of 0.1. For comparison purposes, LR was also implemented as a standard baseline. The RF and SVR models were designed using ensemble learning and the kernel trick, respectively, to support the different experimental datasets. Deep learning architectures underwent 100 iterations of optimisation (batch size = 8, verbose = 0), whereas traditional ML algorithms were trained using default hyperparameter settings. The architectures of the models employed are shown in Table 5.

**Figure 17:**
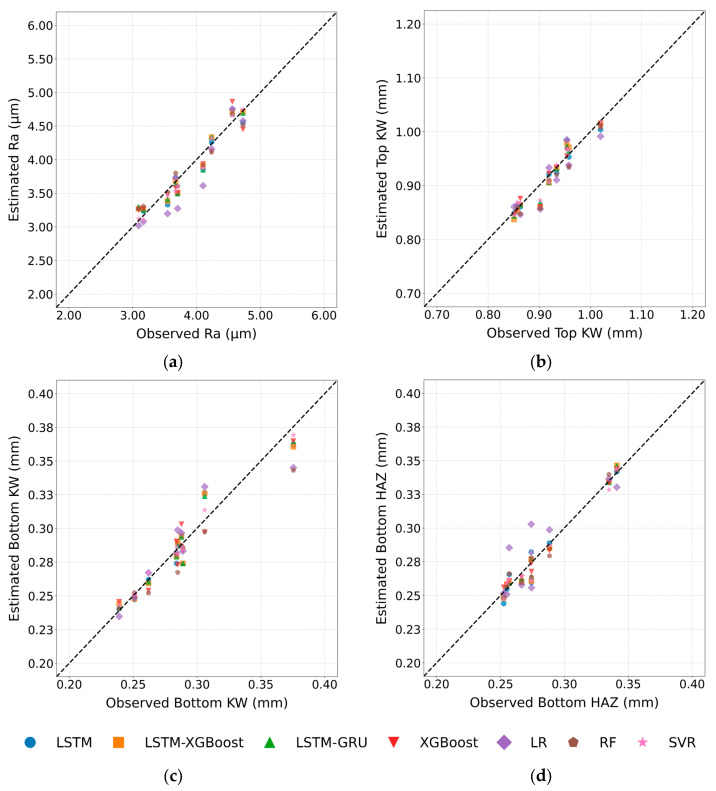
Estimated observed graphs; (**a**) for Ra, (**b**) for Top KW, (**c**) for Bottom KW, (**d**) for Bottom HAZ.

This comparative analysis laid the groundwork for evaluating the models’ predictive performance on Ra, µm, Top and Bottom KW, and Bottom HAZ, which are critical indicators of laser cutting quality. Figure 17 presents scatter plots comparing the observed and predicted values for these response variables. For Ra, the data points from the LSTM–GRU and LSTM–XGBoost models cluster tightly along the 45° line, confirming their low RMSE values, whereas the LR results show noticeable scatter below the diagonal. In the Top KW plot, the XGBoost predictions lie almost exactly on the diagonal, illustrating its clear dominance; LSTM–XGBoost and RF also stay close to the ideal line, while LR again performs poorly. For Bottom KW, the LSTM–GRU, SVR, and LSTM–XGBoost show a close alignment with the diagonal as the models share nearly identical and low error magnitudes. The Bottom HAZ further indicates LSTM–GRU with RF showing the least deviation from the diagonal, whereas LR recorded the farthest spread. All four scatterplots, therefore, provide evidence for the conclusion drawn in the table of metrics: hybrid deep learning models (LSTM–GRU, LSTM–XGBoost) and ensemble models (XGBoost, RF, SVR) offer strong predictive accuracy, whereas LR consistently underperforms.

**Figure 18:**
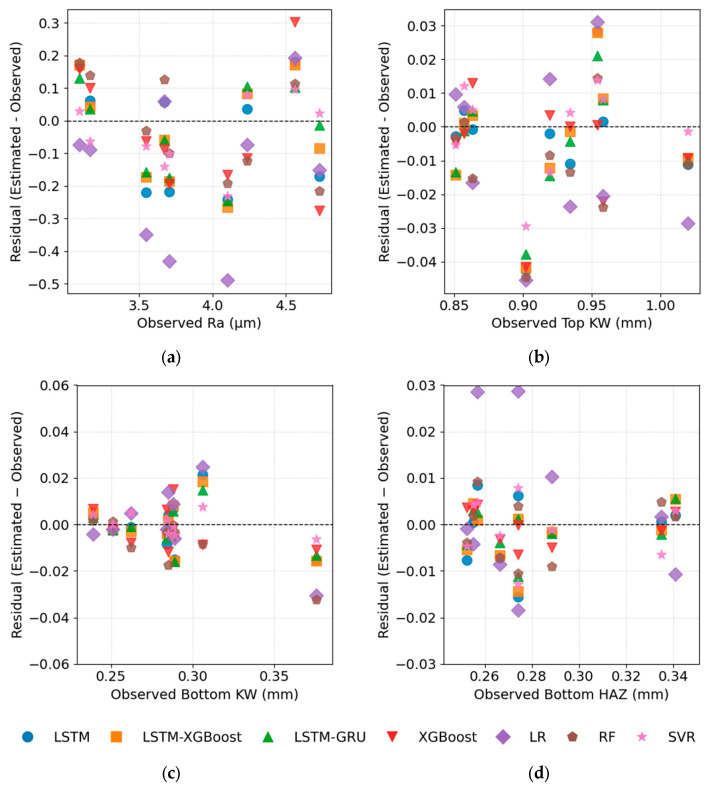
Residual analysis; (**a**) for Ra, (**b**) for Top KW, (**c**) for Bottom KW, (**d**) for Bottom HAZ.

Figure 18 presents a residual analysis comparing the observed and predicted values for the response variables. The residual plots across the four panels further corroborate the quantitative metrics: for Ra, the residuals from the LSTM–GRU model are concentrated within ±0.10 µm of zero, and XGBoost shows a similarly tight distribution, while LR produces the largest negative outliers (≈−0.50 µm), highlighting its bias and poor fit. For Top KW, the XGBoost residuals remain entirely within ±0.015 mm. In contrast, LR—and an unlabelled grey series—display the widest spread, ranging from approximately −0.04 mm to +0.03 mm, indicating systematic under-prediction and occasional over-prediction. For Bottom KW, the residuals from LSTM–GRU, SVR, and LSTM–XGBoost cluster symmetrically within ±0.010 mm of the ideal line, while LR again exhibits the largest negative deviations (≈−0.03 mm). In the Bottom HAZ panel, the smallest residuals are observed for LSTM–GRU and LSTM–XGBoost, remaining mostly within ±0.005 mm. By contrast, LR—and, to a lesser extent, Random Forest (RF)—exhibit a consistent positive bias of approximately +0.02 to +0.03 mm. Overall, the near-zero, homoscedastic residual distributions of the hybrid DL and ensemble models (LSTM–GRU, LSTM–XGBoost, XGBoost, RF, SVR) confirm their superior predictive accuracy, whereas LR consistently exhibits both bias and higher dispersion across all four quality metrics.

**Figure 19:**
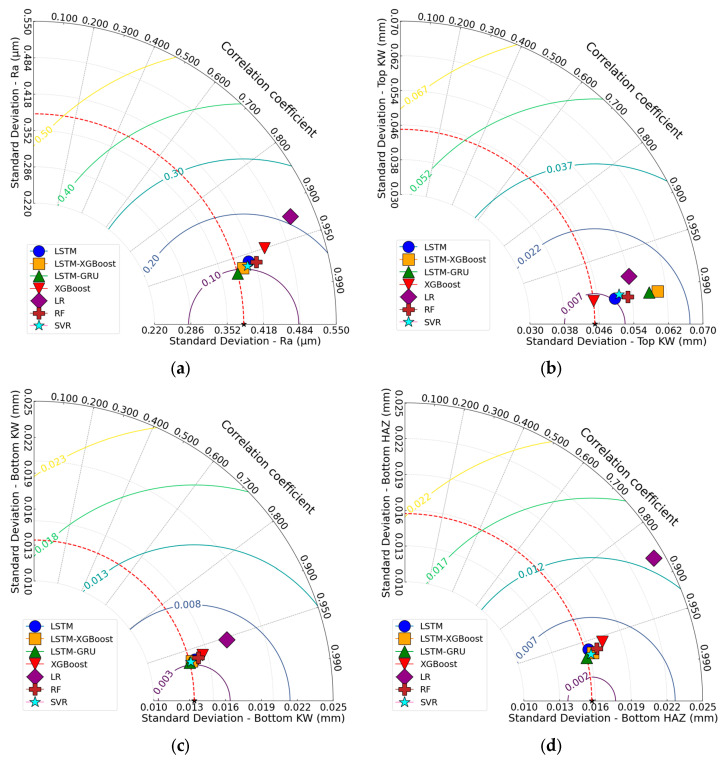
Taylor diagrams; (**a**) for Ra, (**b**) for Top KW, (**c**) for Bottom KW, (**d**) for Bottom HAZ.

Figure 19 presents Taylor diagrams comparing the performance of the different models. For Ra, the LSTM-GRU, LSTM-XGBoost, and SVR points lie almost exactly on the unit standard deviation circle (σ ≈ 0.40 µm), with correlation coefficients of r ≈ 0.97, indicating the lowest centred RMS error. LR, by contrast, exhibits both a higher variance (σ ≈ 0.51 µm) and a lower correlation (r ≈ 0.92), revealing its model bias. For Top KW, XGBoost is closest to the reference point (σ ≈ σ_ref = 0.05 mm, r ≈ 0.99), while LSTM-XGBoost and RF cluster nearby with similarly low error. LR diverges markedly, showing greater variance (σ ≈ 0.054 mm) and reduced correlation (r ≈ 0.98). For Bottom KW, LSTM-GRU, SVR, and LSTM-XGBoost form a tight cluster within the σ ≈ 0.013–0.014 mm range and r ≈ 0.97. LR, however, falls outside the 0.02 mm centred RMSE contour, with σ ≈ 0.017 mm and r ≈ 0.95. For Bottom HAZ, LSTM-GRU is closest to the reference point; XGBoost and SVR follow, with correlations up to r ≈ 0.97 and σ ≈ σ_ref (≈0.016 mm). LR (σ ≈ 0.024 mm, r ≈ 0.87) and, to a lesser extent, RF, exhibit a larger error radius and a positive bias. In general, ensemble and hybrid models of deep learning (LSTM-GRU, LSTM-XGBoost, XGBoost, RF, SVR) carry with them high correlation and a near-unit standard deviation, yielding the minimum centred RMS error, whereas LR consistently performs poorly with high variances and weaker correlation along all four quality indexes under consideration.

Table 6 presents performance metrics and extracted features to judge the predictive accuracy of each model. These metrics were stated as follows: MSE, MAE, RMSE, R^2^, and Pearson correlation, collectively measuring an error magnitude and statistical fit. Performance metrics and features of the ML models are presented in Table 6. For Ra, the LSTM-GRU model performs best (MSE: 0.0391, R^2^: 0.942), while LR shows the weakest performance (MSE: 0.0965, R^2^: 0.85). For Top KW, XGBoost slightly outperforms the other models (MSE: 0.00034, R^2^: 0.987), whereas LR yields the highest error (MSE: 0.0011). For Bottom KW, LSTM-GRU again leads (MSE: 0.000179, R^2^: 0.943), while LR lags behind (MSE: 0.000287, R^2^: 0.909). For Bottom HAZ, LSTM-GRU achieves the best results (MSE: 0.000198, R^2^: 0.947), whereas LR performs poorly (MSE: 0.000912, R^2^: 0.753). The LSTM-GRU hybrid model consistently demonstrates the best performance across all output parameters (Ra, Top KW, Bottom KW, Bottom HAZ), achieving the lowest errors (MSE, MAE, RMSE) and the highest R^2^ and Pearson correlation scores, indicating superior predictive accuracy and robustness compared to the other models. The LSTM-GRU, with its hybrid architecture that has long-term memory capacity combined with gating mechanisms of high efficiency, outclassed all other models. Hence, it successfully captures highly complex nonlinear relations in the laser cutting data more efficiently than any other model.

## 5. Conclusions

This paper systematically evaluated Fused Filament Fabrication (FFF)-printed ABS thermoplastics during laser cutting with experimental and machine learning methods. It places special attention on three critical input parameters (cutter velocity, power, and workpiece thickness) and four output variables (surface roughness (Ra), top kerf width (Top KW), bottom kerf width (Bottom KW), and bottom heat–affected zone (Bottom HAZ)) in the studies. A total of 45 experiments were performed with CO_2_ laser cutting under controlled conditions. The data were then treated using statistical evaluation through ANOVA and computational methods via several ML models for predicting laser-cutting quality output parameters from process parameters.

Experimental results identified cutting speed as the most influential parameter across all quality metrics, significantly improving surface finish, kerf width, and heat-affected zone by reducing laser–material interaction time. Laser power showed secondary effects, especially at low speeds, while plate thickness had a limited but contextual impact. ANOVA results supported these findings, with cutting speed explaining up to 92% of the variation in Bottom HAZ. All models demonstrated strong reliability, with R^2^ values exceeding 90%. Seven machine learning models were evaluated to predict laser cutting quality based on process parameters. Among them, the LSTM-GRU hybrid model achieved the highest accuracy, with the lowest MSE and highest R^2^ and Pearson values across all outputs. Graphical analyses further confirmed the superiority of hybrid deep learning and ensemble models (LSTM-GRU, LSTM-XGBoost, XGBoost), while traditional models like LR showed notably weaker performance.

The integration employed in this study combines experimental evaluations with predictive modelling, making it relevant to both practitioners and theorists. Practically, such an integration allows the selection and fine-tuning of CO_2_ laser cutting parameters onto ABS parts manufactured by FFF with a higher level of understanding, thereby lessening the costs in trial-and-error experimentation. Theoretically, the synergy of the various approaches adds to the already vast literature base of Artificial Intelligence applications in the context of AM and post-processing. Real-life 3D-printed ABS product manufacturing can easily put into practice the results of this research to enable manufacturers to have the laser cutting parameters optimised, especially cutting speed, for the best surface quality and dimensional tolerance. Besides, the developed machine learning models, notably the LSTM-GRU model, may well act as predictive tools in digital manufacturing systems to limit the trial-and-error processes whilst improving the overall efficiency of the process. It also fills a considerable void in the literature by concentrating on ABS material less commonly studied in the laser cutting predictive modelling context. The prevailing literature often centres on PLA or PETG, while very few combine experimental and machine-learning-based approaches for ABS, especially utilising a more advanced hybrid deep learning architecture. For potential future work, some suggestions include: increasing the number of samples and parameter ranges within the dataset to make model generalisation even better, incorporating other quality characteristics (kerf taper, material removal rate, or microstructural changes), integrating real-time process monitoring data (thermal imaging, laser reflection, acoustic signals, etc.) into ML models for adaptive process control support, and using transfer learning methods to extend models trained on ABS to other thermoplastics or composite materials. Future work will aim to investigate a wider range of laser powers and additional process parameters, such as pulse frequency and assist gas pressure, to further enhance model robustness and cutting performance insights.

In conclusion, the study highlighted that performing experimental trials coupled with data-driven modelling, especially those relying on hybrid deep learning algorithms, can play a significant role in the accurate prediction and optimisation of CO_2_ laser cutting performance on FFF-printed ABS thermoplastics. This approach could serve as a means for intelligent, efficient, and high-precision post-processing toward this now marvellous domain of polymer-based AM.

## Figures and Tables

**Table 1:** Technical specifications of ABS.

Properties	Value
Manufacturer	Filameon
Filament	ABS
Print temperature (°C)	230–260
Diameter (mm)	1.75
Density (g/cm^3^)	1.04
Tensile strength (MPa)	45
Elongation (%)	10
Flexural strength (MPa)	73
Rockwell hardness (R scale)	108
Glass transition temperature (°C)	85
Melt flow index (220 °C/10 kg)	23

**Table 2:** Three-dimensional printing settings used for the manufacturing of the test specimens.

Parameter	Value
Printing orientation (Degree)	±45
Layer thickness (mm)	0.24
Bed temperature (°C)	100
Extrusion temperature (°C)	250
Infill pattern	line
Wall line count	3
Top solid layer	5
Bottom solid layer	4
Fill density (%)	100
Printing speed (mm/s)	40
Fan speed	100

**Table 3:** Factors and levels used in the laser cutting process.

Factors	Level				
	1	2	3	4	5
Plate thickness (mm)	2	2.5	3	3.5	4
Cutting speed (mm/s)	3	6	9	-	-
Power (W)	87.5	92.5	97.5	-	-

**Table 4:** ANOVA results for Ra, Top KW, Bottom KW, and Bottom HAZ.

Source	DF	Seq SS	Adj SS	Adj MS	F-Value	*p*-Value	Contribution (%)
**Ra (µm)**							
Plate thickness (mm)	4	2.0414	2.0414	0.51035	26.37	*p* < 0.001	12.25
Power (W)	2	2.4072	2.4072	1.20358	62.20	*p* < 0.001	14.45
Cutting speed (mm/s)	2	11.5144	11.5144	5.75721	297.52	*p* < 0.001	69.12
Error	36	0.6966	0.6966	0.01935			4.18
Total	44	16.6596					100
R^2^ = 95.82%, R^2^ (adj) = 94.89%, R^2^ (pred) = 93.47%							
**Top KW (mm)**							
Plate thickness (mm)	4	0.020361	0.020361	0.005090	93.86	*p* < 0.001	10.99
Power (W)	2	0.011908	0.011908	0.005954	109.78	*p* < 0.001	6.43
Cutting speed (mm/s)	2	0.150989	0.150989	0.075495	1392.00	*p* < 0.001	81.52
Error	36	0.001952	0.001952	0.000054			1.05
Total	44	0.185211					100
R^2^ = 98.95%, R^2^ (adj) = 98.71%, R^2^ (pred) = 98.35%							
**Bottom KW (mm)**							
Plate thickness (mm)	4	0.025151	0.025151	0.006288	62.82	*p* < 0.001	35.99
Power (W)	2	0.008082	0.008082	0.004041	40.37	*p* < 0.001	11.56
Cutting speed (mm/s)	2	0.033052	0.033052	0.016526	165.10	*p* < 0.001	47.29
Error	36	0.003604	0.003604	0.000100			5.16
Total	44	0.069888					100
R^2^ = 94.84%, R^2^ (adj) = 93.70%, R^2^ (pred) = 91.94%							
**Bottom HAZ (mm)**							
Plate thickness (mm)	4	0.002301	0.002301	0.000575	33.33	*p* < 0.001	3.51
Power (W)	2	0.002291	0.002291	0.001146	66.37	*p* < 0.001	3.50
Cutting speed (mm/s)	2	0.060266	0.060266	0.030133	1745.89	*p* < 0.001	92.04
Error	36	0.000621	0.000621	0.000017			0.95
Total	44	0.065480					100
R^2^ = 99.05%, R^2^ (adj) = 98.84%, R^2^ (pred) = 95.52%							

**Table 5:** Architecture of the models used.

Model	Architecture	Optimisation
LSTM	Single layer with 64 neurons	Adam (LR = 0.001)
LSTM-XGBoost (Hybrid)	LSTM (32 units) + Dense (16 units)	Adam (LR = 0.001)
LSTM-GRU (Hybrid)	LSTM (32 units) + GRU (32 units)	Adam (LR = 0.001)
XGBoost	100 trees, learning_rate = 0.1	Gradient Boosting
LR	Least squares method	–
RF	100 decision trees	Bootstrap Aggregating (Bagging)
SVR	RBF kernel, C = 100, gamma = 0.1	Kernel Trick

**Table 6:** Performance metrics for different models and features.

Feature	Model	MSE	MAE	RMSE	R^2^	Pearson
Ra (µm)	LSTM	0.052600	0.193600	0.229300	0.921000	0.960000
	LSTM-XGBoost	0.044900	0.178900	0.211900	0.933000	0.966000
	**LSTM-GRU**	**0.039100**	**0.167200**	**0.197700**	**0.942000**	**0.971000**
	XGBoost	0.063200	0.213400	0.251400	0.902000	0.950000
	LR	0.096500	0.263800	0.310600	0.850000	0.922000
	RF	0.048300	0.185500	0.219800	0.928000	0.963000
	SVR	0.045800	0.180300	0.214000	0.931000	0.965000
Top KW (mm)	LSTM	0.000380	0.015600	0.019500	0.986000	0.993000
	LSTM-XGBoost	0.000450	0.017200	0.021200	0.983000	0.992000
	LSTM-GRU	0.000420	0.016500	0.020500	0.984000	0.992000
	**XGBoost**	**0.000340**	**0.014800**	**0.018400**	**0.987000**	**0.994000**
	LR	0.001100	0.027600	0.033200	0.958000	0.979000
	RF	0.000390	0.016000	0.019700	0.985000	0.993000
	SVR	0.000470	0.017800	0.021700	0.982000	0.991000
Bottom KW (mm)	LSTM	0.000193	0.011845	0.013899	0.938421	0.968724
	LSTM-XGBoost	0.000185	0.011623	0.013607	0.940982	0.970045
	**LSTM-GRU**	**0.000179**	**0.011412**	**0.013384**	**0.943012**	**0.971096**
	XGBoost	0.000214	0.012462	0.014632	0.931845	0.965308
	LR	0.000287	0.014438	0.016941	0.908523	0.953158
	RF	0.000201	0.012036	0.014178	0.935672	0.967302
	SVR	0.000183	0.011539	0.013527	0.942157	0.970651
Bottom HAZ (mm)	LSTM	0.000267	0.013700	0.016300	0.928000	0.963000
	LSTM-XGBoost	0.000216	0.012300	0.014700	0.942000	0.971000
	**LSTM-GRU**	**0.000198**	**0.011700**	**0.014100**	**0.947000**	**0.973000**
	XGBoost	0.000289	0.014500	0.017000	0.922000	0.960000
	LR	0.000912	0.025800	0.030200	0.753000	0.868000
	RF	0.000242	0.013000	0.015600	0.935000	0.967000
	SVR	0.000219	0.012400	0.014800	0.941000	0.970000

## Data Availability

Data are contained within this article.
